# A qualitative study of enablers and barriers influencing the incorporation of social accountability values into organisational culture: a perspective from two medical schools

**DOI:** 10.1186/s13584-015-0044-5

**Published:** 2015-12-10

**Authors:** Nicholas Leigh-Hunt, Laura Stroud, Deborah Murdoch Eaton, Mary Rudolf

**Affiliations:** Division of Primary Care and Public Health, Leeds Institute of Health Sciences, Faculty of Medicine and Health, Leeds University, Charles Thackrah Building, 101 Clarendon Road, Leeds, LS2 9LJ UK; The Medical School, University of Sheffield, Beech Hill Road, Sheffield, S10 2RX UK; Faculty of Medicine in the Galilee, Bar Ilan University, Ramat Gan, 5290002 Israel

**Keywords:** Social accountability, Social responsibility, Social mission, Medical school, Enablers, Barriers, Organisational change

## Abstract

**Background:**

Definitions of social accountability describe the obligation of medical schools to direct education, research and service activities towards addressing the priority health concerns of the population they serve. While such statements give some direction as to how the goal might be reached, it does not identify what factors might facilitate or hinder its achievement. This study set out to identify and explore enablers and barriers influencing the incorporation of social accountability values into medical schools.

**Methods:**

Semi structured interviews of fourteen senior staff in Bar Il**a**n and Leeds medical schools were undertaken following a literature review. Participants were recruited by purposive sampling in order to identify factors perceived to play a part in the workings of each institution.

**Results:**

Academic prestige was seen as a key barrier that was dependent on research priorities and student selection. The role of champions was considered to be vital to tackle staff perceptions and facilitate progress. Including practical community experience for students was felt to be a relevant way in which the curriculum could be designed through engagement with local partners.

**Conclusions:**

Successful adoption of social accountability values requires addressing concerns around potential negative impacts on academic prestige and standards. Identifying and supporting credible social accountability champions to disseminate the values throughout research and education departments in medical and other faculties is also necessary, including mapping onto existing work streams and research agendas. Demonstrating the contribution the institution can make to local health improvement and regional development by a consideration of its economic footprint may also be valuable.

## Background

Definitions of social accountability describe the obligations of medical schools to direct their education, research and service activities towards addressing the priority health concerns of their populations [1]. While such statements give some direction as to how the goal might be reached, it does not identify what factors might facilitate or hinder its achievement.

These factors could potentially be external, such as the prevailing political climate, the economic situation, or the structure of the health services in the region or country in which the medical school is sited; they could be more closely related to the institution, such as the staff, students, curriculum or the community in which it is located. For example, one factor allowing an academic institution to become more socially accountable would be genuine involvement of the local community in the design and delivery of both research and educational activities [2–4], through patient participation groups and curriculum review committees. Selecting students from the local community, or from other underserved areas, through the use of preferential admission policies, could be another means for the institution to demonstrate it is engaging with its locality [5, 6]. However the path may not be straightforward and the organisational culture of a medical school may obstruct intentions, presenting a major barrier to making progress, with the result that educational activities and the research agenda neither reflect local priorities nor are aligned with social accountability values [7, 8]. This may be more than a staff issue, as students may also fail to see the relevance of social accountability to them personally [9, 10]. Funding too could be an issue, with the lack of resources preventing related projects getting off the ground or the nature of the funding leading to research that is unreflective of local priorities [11, 12]. However it is not clear how many of these factors are truly relevant, to what extent they have an impact or how they may interact.

A literature search indicated that there is some discussion of the barriers and levers that may influence the adoption of social accountability values in programs from around the world, though no studies have specifically set out to identify them. We therefore undertook this study to explore the factors that influence the adoption of social accountability values within an organisation. We carried out the study in two medical schools at different stages of development: Leeds University Medical School in the United Kingdom, established in 1831 and Bar Ilan Medical School in Israel, founded in 2011.

## Methods

A qualitative study design was chosen to explore the beliefs, opinions and perceptions of staff in the medical school regarding social accountability as defined by the World Health Organisation to gain an insight into the workings of the medical school at a strategic level. Prior to the start of the research, ethical approval was gained via the Leeds University Faculty of Medicine and Health Joint Research Ethics Committee. Research was undertaken in Bar Ilan Medical School (BI) in Safed in Israel and Leeds University Medical School (UoL). These two medical schools were chosen as it was considered they would provide a spectrum of views and opinions for the study, since one is an established institution with nearly two centuries of tradition while the other was founded only very recently; both institutions are primarily state funded with additional charitable funding. Social accountability has been in the mission statement since the founding of Bar Ilan medical school, and while not in the founding charter for Leeds it was recently adopted by the School of Medicine Student Educational Strategy as a core value. Both medical schools have clearly stated strategy intended to embed social accountability principles within their schools, and are due to undertake evaluation of their progress towards this.

Purposive sampling was used to recruit potential study participants from a small pool of senior individuals responsible for strategy and leadership within the two medical schools. These included the medical school deans, the deans for preclinical and clinical medical education, heads of faculties of public health, research deans, other clinicians, finance directors and a chief executive. Participants were initially approached by MR and DME, senior academics and members of the research team at each site. Participant information was circulated to potential participants and consent was gained at the time of interview. Between six and eight individuals in each medical school were recruited ([BI X] = Bar Ilan interviewee X, [UoL Y] = Leeds University interviewee Y) reflecting the number of individuals who were in a position to identify the factors that influence the incorporation of social accountability values within the workings of the medical school and who had a role in implementing strategy.

**Fig. 1:**
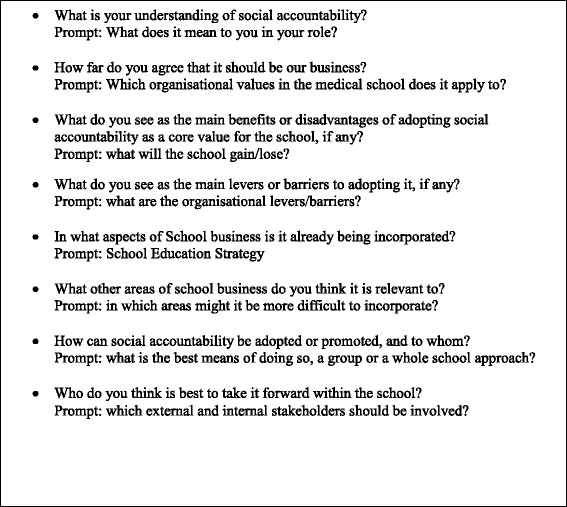
Topic guide

Semi-structured interviews, lasting no more than an hour, were conducted in English in both countries. A topic guide (Fig. 1) containing question prompts was used to guide interviews; the interviewees were able to speak freely on the subject rather than answering a set list of questions. Participants were made aware that full anonymity may not be possible because of the small number of potential respondents, though they were informed that the use of direct quotes in the report would be avoided to minimise this.

Thematic analysis was used to analyse the transcripts of the interviews [13, 14]. Common themes were identified from preliminary analysis of the interviews and codes defined; codes were then assigned to the text of each transcript, and used as a basis for writing up the findings.

## Results and discussion

The main themes that were identified in the interviews were similar at both sites and could be categorised into: those relating to institutional systems and staff, such as academic prestige, personal staff issues, research priorities and delivery; those relating to students, such as selection processes and student values; those relating to curriculum design and delivery; externally related factors such as stakeholder partnerships and the economic footprint; and finally those related to the process of implementing social accountability itself, such as the dissemination strategy and evaluation of progress. There were small differences between the two schools on: the population that should be used as a reference point to which the school should be accountable, partly influenced by the number of international students and overseas projects; the type of research projects that could be defined as being socially accountable, also dependent on to their funding sources; and the need to invest in the institution and retain graduates. The enablers and barriers identified are summarised in Table 1.

**Table 1:** Barriers and enablers to implementing social accountability

	Barriers	Enablers
External factors	Economic instability	Government/funders expectation of accountability
	Potential instability in partner organisations	Effective partnerships especially with voluntary organisations
		Economic contribution to regional development and local health improvement
Institutional systems & staff	Emphasis on maintaining academic prestige	Good communication between the institution and partners
	Success defined in terms of degree results rankings and graduates becoming tertiary specialists	Emphasis on advocacy and enabling communities to advocate for themselves
	Staff personal time pressures, political views, level of interest, conceptual understanding and commitment	
Research priorities, design, delivery	Emphasis on laboratory research	Patient and public participation with grassroots developed projects
	Need to ensure financial viability of research departments	
		Explicit requirement to identify patient benefit in research proposals
	Social accountability viewed as a distraction	
	Source of funding	Emphasis on translational research
		Support from regional health authorities
Student selection & values	Widening participation seen as detrimental to prestige	Targeted support to students from underrepresented backgrounds
	Difficulty of selecting students on their values	
	Change in student values over time in education	Recruiting internationally
		Graduate retention
Curriculum design & delivery	Narrow focus of curriculum on clinical skills and procedures	Teaching on wider heath determinants and communities
		Involvement of students in community projects or, voluntary work
	Uncertainty of geographical location for which students should be trained	
		Empowering students to challenge other health professionals
	Relative newness of the concept	Auditing of outcomes of such placements and providing
		Adequate support to students in external placements
Implementation & evaluation	Difficulty of developing metrics to gauge progress	Presence of fully supported champions
	Assessment fatigue	Demonstrating the impact of the institution via assessment
		Availability of guidance
		Assessment as a driver of change

### Academic prestige

Nearly all participants noted academic prestige as an issue, and how this could conflict with social accountability values in both education and research, particularly if success was primarily defined in terms of laboratory research and ranking highly in terms of degree results. “*Medical schools…still see their successful students as those that become tertiary care specialists doing cutting edge research, principally lab research*” [BI 2]. Prestige was viewed as important to attract staff and students. “*If you’re seen as a faculty with lowered standards then you attract lower standard students…lower standard staff*” [BI 1]. There was recognition that social accountability could be seen as a distraction and adopting it might lead to diversion of funding from laboratory research. One of the key drivers behind these concerns was the need to ensure financial viability of education or research departments, though participants were not oblivious to the potential impact of the source of funding. “*I’d be very careful about where the funding comes from…especially if it contradicts what your values are”* [UoL 7].

### Staff attitudes and personal issues

Other factors perceived as important were staff personal time pressures, political views, level of interest, awareness or commitment. Many thought that there were barriers in terms of the variation in conceptual understanding and to whom the institution should be accountable. “*Its not necessarily that there’s one view as to what social accountability is, the question is does it mean all things to all people*” [UoL 3], “*When you talk about social accountability, it’s not entirely clear who the medical school is accountable to*” [BI 2].

### Strategy dissemination

It was suggested that these views could be challenged by highlighting the values in the organisational strategy and demonstrating how the institution was fulfilling its purpose through both education and research. “*The government increasingly tells universities that they have to be accountable to the people who are funding them*” [UoL 1], “*That was absolutely built into the remit of the faculty…to demonstrate in what way we are impacting on the region”* [BI 1]. The role of champions in circumventing barriers was also acknowledged”*There need to be champions who will drive this forward because otherwise…social accountability might have dropped out of the agenda so somebody’s got to be keeping it alive in people’s minds*” [UoL 5]. Such champions would need to be fully supported and know when to delegate. “*It’s a school mission, it’s not the mission of this person, this department*” [BI 5], “*In any major new initiative you need champions and there needs to be the passion to make it work… but if the champions are good they don’t do it all themselves*” [BI 2].

### Research priorities and delivery

Other useful levers mentioned were the requirement to identify patient benefit when submitting research proposals and favouring translational research, with strong support from regional health authorities for such projects. Fully involving the community in research design was seen as important regardless of any difficulties such as translating research into practice or the expense of dissemination. “*The fact that funders have sent out a very clear message that … patients and public participation is important…has led to some quite active engagement with local communities”* [UoL 5].

### Evaluation of progress

Evaluation was thought to be another important lever provided it was neither a tick box exercise nor an exhaustive process. “*Assessment always focuses action, action that takes place in an environment without assessment tends to be less focussed…I think the act of assessment changes the action itself”* [BI 4], “*There does need to be a process in place where medical schools demonstrate their social accountability”* [BI 2]. The difficulty of developing metrics to gauge progress was seen as problematic especially when projects involved more than one organisation “*It’s very difficult to quantify social accountability or come up with any measures that categorically indicate that you’re at the right end of the spectrum or not*” [UoL 3]. The availability of existing guidance was seen as being helpful, including that from medical regulatory authorities. “*How do you evaluate social accountability, thank goodness there are a few frameworks, there is ASPIRE, there is WHO*” [BI 7].

### Student selection

The role of the student selection process was discussed with local recruitment being seen as relevant to social accountability, though doubts were expressed whether locally recruited applicants would remain in the local area after graduation. Concerns were raised about potential adverse impacts on academic prestige “*A widening participation agenda where you might offer lower grades might affect rankings*” [UoL 7]. Others thought targeted support to students from underrepresented backgrounds in the form of bursaries and employment offers in socially useful work a better strategy. . “*We’ll do everything we can…to help students…from needy backgrounds achieve, but as far as we’re concerned the entrance to the medical school is done on a level playing field*” [BI 1]. Recruiting internationally was thought to be vital “*The international staff and the international students…I think utilising their cultural experiences to challenge students perceptions are absolutely invaluable*” [UoL 2].

### Student values

Selecting students on their values was viewed as equally important for social accountability but problematic “*There’s a huge amount of debate going on all the time about methods of selecting students and how accurate can you be, in judging a character and intentions and potential*” [BI 4]. Others were concerned that even when students were selected on the basis of their values, the latter could change during the educational process. “*Students come in with positive attitudes that just get knocked out of them, traditionally gets knocked out of them by being in medical school*” [BI 2]. A good means of countering this was felt to be involving students in practical community projects, voluntary work or empowering them to challenge other health professionals. “*Empowering them so that they can and do question and know lines of reporting, and know when they should walk away from situations that may be ethically inappropriate*” [UoL 2].

### Curriculum design

Social accountability values were seen as inherently relevant to medical education. “*Medical schools are probably quite lucky in the sense that probably most of what we understand by social accountability, we’d recognise in medicine*” [UoL 7]. However when designing the curriculum there were differing opinions as to whether this related to specific skills and procedures or ensuring future doctors had a wider understanding of health and communities. Another perceived barrier was the geographical location students should be trained for. “*There’s probably a bit of tension in what I might see as wider social accountability… preparing students to serve a global population if you like and the tension that really we’re funded by the Department of Health to produce doctors for the UK*” [UoL 4]. The newness of the concept for those designing the curriculum was seen as a further barrier. “*Social accountability is such a very new concept in its current form that the people designing the curricula for the individual courses aren’t au fait with it*” [BI 2].

### Curriculum delivery

Participants felt that practical community experience was important given that healthcare in future was more likely to occur outside of hospitals, especially if students found they could make more of a difference with a community based project than a hospital audit. Acknowledging the contribution of voluntary organisations for this purpose was seen as essential. “*A lot of the voluntary sector organisations provide placements for the students for an absolute minimal sum, it’s really important that…it’s not at cost to the voluntary organisation…it is a partnership and one would hope that the students working with the organisation would produce something that’s of value to the organisation”* [UoL 2]. Auditing the outcomes of such placements and providing adequate support to students in them was another requirement. “*They have to feel that it’s not something that we just send them on and forget them and they are being left alone to battle with the placements”* [BI 7].

### Stakeholder partnerships

Having effective partnerships was viewed as essential to making progress in being socially accountable, particularly when mutual gains could be identified and partnership projects were developed from the grassroots. Going beyond giving communities a voice by enabling them to develop a voice was seen to be of high importance. “*There’s a course here for community activists…set up for community activists interested in narrowing the health divide”* [BI 1]. The value of good communication between the institution and partners was recognised, through having either a patient and community forum or a dedicated community contact, though it was acknowledged that internal dialogue within the institution could limit this. However potential instability in partner organisations was seen as an obstacle. “*The embracing of this mission of education will very much depend upon the stability of the organisation in which the students are sent for their placement*” [UoL 3]. Others considered this not to be a problematic as the overriding mission would remain the same or because change could be a positive force. “*Sometimes organisational change is required because the external environment has changed so much that if the organisation doesn’t change then it can’t deal with the new pressures*” [UoL 7].

### Economic footprint

Economic instability was seen as being relevant, which could be seen either positively or negatively. “*The recession of course has its impact on the society and may mean that society expects more of universities, to be more cost effective and to deliver more”* [UoL 1]. It was also felt necessary to be aware of the economic footprint of the medical school and its potential beneficial impact on the local economy. “*What makes the university successful…in a sense stimulates the local economy*” [UoL 7], “*It’s been said by many…politicians, public figures around the area that the faculty is a huge engine for development*” [BI 4]. The institution was considered to be inherently obliged to play a developmental role. “*We have an obligation to the people around us…it could be in ways of perhaps helping to strengthen human infrastructure in the area, perhaps even bringing economic benefits*” [BI 4].

## Conclusion

This study reveals some important findings as regards implementing social accountability though findings may not be entirely generalisable; while they were founded nearly two centuries apart in different countries, only two medical schools were involved in this study. It may also be argued that Leeds Medical School is reasonably representative of medical schools in the United Kingdom but that Bar Ilan Medical School being so new, may be atypical of those in Israel. Furthermore as both Leeds and Bar Ilan Medical Schools have encountered difficulties in implementing social accountability despite making it a priority goal, the challenges are likely to be even greater in those medical schools which have not done so explicitly.

While there are an increasing number of reports in the literature of efforts directed towards making medical schools more socially accountable, our research provides the first report about levers and barriers to implementation. Our findings relate to concerns regarding academic prestige, the need to identify champions, providing community exposure and demonstrating potential contributions to the locality. Understanding these obstacles and facilitators are essential to taking the social accountability agenda forward.

Academic prestige appeared to be a major obstacle to implementing social accountability, with concerns that doing so could adversely affect status in terms of both education and research, with a detrimental impact on the ability to attract grants, staff and students of sufficient calibre, or potentially diverting funds from laboratory based research; adopting its values may be seen as lowering standards for students, both at entry into medical school and during their education there. Therefore it may be necessary to show that adopting the values will enhance prestige in two main ways.

Firstly, if more graduates have the appropriate set of professional values, it will allow the institution to demonstrate that it is better fulfilling its purpose to taxpayers and enhance its reputation especially in the light of recent scandals around standards in healthcare [6, 15]. Encouraging applicants from underrepresented backgrounds needs to be recognised as allowing the institution to be viewed positively; academic standards can be maintained while doing this if preferential admission policies are avoided and outreach programmes used instead, together with financial and psychological support for such students [16].

Secondly, the institution will be able to show how it is making an appreciable difference to the local community through health improvement, by opting for translational research and ensuring patient and public participation. This will allow academics to continue to realise personal career aspirations, and overtly demonstrate that adopting social accountability will not be done as a substitute for undertaking laboratory research or to placate the more strident demands of some community representatives. However to facilitate this negative perceptions in the community concerning the institution may need to be overcome, and communication and knowledge gaps may need to be rectified [5, 10].

Identifying where social accountability maps onto existing work streams may ensure greater commitment of staff through recognition of work they may have already undertaken; identifying potential financial incentives may address concerns around time pressures, priorities, and resources. Fully supported social accountability champions will be essential to assist with this process, especially to prevent social accountability being seen as the responsibility of solely medical education or public health departments [17] and to disseminate social accountability values throughout the university beyond medical faculties. Champions will need to tackle misunderstandings around the concept, especially as many will be new or unfamiliar with it, by making use of existing guidance and good practice examples on websites [18].

Evaluation of progress should serve to focus action but should not be exhaustive. Each institution should develop its own indicators of progress as a uniform approach for all medical schools is unlikely to be suitable. Above all else evaluation should be able to transparently demonstrate the difference made to local communities, particularly by students. Concerns around a perceived lack of clarity around performance indicators and the absence of financial incentives to meet them [10] will need to be addressed by highlighting the number of sources that can be used to derive a suitable variety of metrics, such as the THEnet evaluation framework [19], ASPIRE [20] documentation and use of the Social Mission Score [21].

Curriculum design and delivery should involve a wide range of community partners, in order to facilitate the education of adaptable, caring and competent practitioners who will be capable of working in different environments, and avoid a narrow focus on the acquisition of clinical skills. Curriculum committees should be truly representative of group interests and not curtailed by informal internal university discussions; they should borrow on the expertise of primary care practitioners who have key local knowledge of health problems.

The concept of social accountability should be introduced early and throughout the curriculum; students need to be engaged through community placements and projects to ensure they have an understanding of the wider determinants of health and are enabled to see social accountability is as much their responsibility as that of the institution. Such placements may serve to counteract the decline in initial enthusiasm that may occur in students as a result of the need for large amounts of fact based learning, but will need to be adequately resourced and regularly audited to assess the quality of learning gained and determine their impact on final career choice [8]. Opportunities for voluntary work should also be fostered, to tap into the altruism of students, with opportunities for paid employment in community placements for less well off students. Empowering students to challenge other health professionals in ethically challenging situations via peer reflective learning sessions may be another means of instilling social accountability values.

**Fig. 2:**
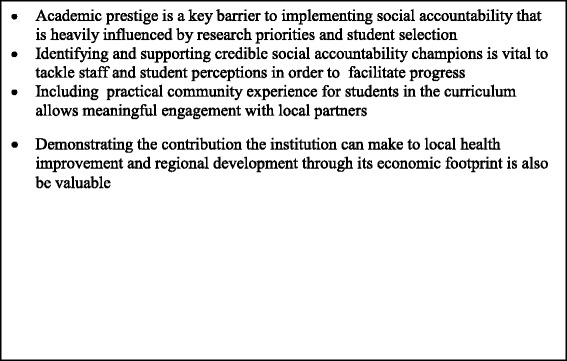
Lessons learnt

The role of the medical school as a major local employer and purchaser should be emphasised and the consequent impact on the local economy through the spending of employees and students considered. This is likely to be especially important when a new medical school is sited in an underdeveloped area or an existing institution plays a role in providing stability in regions where there has been loss of human capital [22] as it will be able to have a key role in regional development. Demonstrating this beneficial aspect of the economic footprint of the institution in addition to demonstrating the real difference through medical education and research should therefore allow the barriers to implementing social accountability to be overcome. Furthermore, as graduates from medical schools may nowadays go on to work anywhere in the world; ensuring social accountability is fully incorporated into their curriculum will guarantee they are equipped with globally transferable skills Fig. 2.

## Abbreviations

BI: Bar Ilan
UoL: Leeds University
